# Split Hand-Foot and Deafness in a Patient with 7q21.13-q21.3 Deletion Not Including the *DLX5/6* Genes

**DOI:** 10.3390/genes14081526

**Published:** 2023-07-26

**Authors:** Irene Ambrosetti, Laura Bernardini, Marzia Pollazzon, Maria Grazia Giuffrida, Valentina Guida, Francesca Peluso, Maria Chiara Baroni, Valeria Polizzi, Manuela Napoli, Simonetta Rosato, Gabriele Trimarchi, Chiara Gelmini, Stefano Giuseppe Caraffi, Anita Wischmeijer, Daniele Frattini, Antonio Novelli, Livia Garavelli

**Affiliations:** 1 Medical Genetics Unit, IRCCS Azienda Ospedaliero-Universitaria di Bologna, 40138 Bologna, Italy; irene.ambrosetti@studio.unibo.it (I.A.);; 2 Clinical Genetics Unit, Azienda USL-IRCCS di Reggio Emilia, 42123 Reggio Emilia, Italy; 3 Division of Medical Genetics, Fondazione IRCCS-Casa Sollievo della Sofferenza, 71013 San Giovanni Rotondo, Italy; 4 Department of Audiology, Azienda USL-IRCCS di Reggio Emilia, 42123 Reggio Emilia, Italy; 5 Neuroradiology Unit, Azienda USL-IRCCS di Reggio Emilia, 42123 Reggio Emilia, Italy; 6 Clinical Genetics Service and Coordination Center for Rare Diseases, Department of Pediatrics, Regional Hospital of Bolzano, 39100 Bolzano, Italy; 7 Child Neurology and Psychiatry Unit, Azienda AUSL-IRCCS di Reggio Emilia, 42123 Reggio Emilia, Italy; 8 Translational Cytogenomics Research Unit, Bambino Gesù Children’s Hospital IRCCS, 00165 Rome, Italy

**Keywords:** ectrodactyly, SHFM, *DLX5*, *DLX6*, *DYNC1I1*, TADs, imprinting, regulatory elements

## Abstract

Split Hand-Foot Malformation (SHFM) is a congenital limb defect characterized by a median cleft of the hands and/or feet due to the absence/hypoplasia of the central rays. It may occur as part of a syndromic condition or as an isolated malformation. The most common of the six genetic loci identified for this condition is correlated to SHFM1 and maps in the 7q21q22 region. SHFM1 is characterized by autosomal dominant transmission, incomplete penetrance and variable expressivity. Associated features often include hearing loss, intellectual disability/developmental delay and craniofacial abnormalities. Disruption of the *DLX5/DLX6* genes, mapping within the SHFM1 locus, is now known to be responsible for the phenotype. Through SNP array, we analyzed a patient affected by SHFM1 associated with deafness and an abnormality of the inner ear (incomplete partition type I); we identified a deletion in 7q21, not involving the *DLX5*/6 genes, but including exons 15 and 17 of *DYNC1I1*, known to act as exonic enhancers (eExons) of the *DLX5*/6 genes. We further demonstrated the role of *DYNC1I1* eExons in regulating *DLX5*/6 expression by means of showing a reduced expression of the *DLX5*/6 genes through RT-PCR in a patient-derived lymphoblastoid cell line. Furthermore, our data and a review of published cases do not support the hypothesis that *DLX5*/6 are imprinted in humans. This work is an example of how the disruption of regulatory elements can be responsible for congenital malformations.

## 1. Introduction

Split hand/foot malformation (SHFM; OMIM 183600), or ectrodactyly, is a congenital malformation of the limbs featuring the median cleft of the hands and/or feet owing to aplasia or hypoplasia of the central metacarpal and/or metatarsal bones and of the phalanges [1]. The prevalence of SHFM is about 1:18,000 live births. It may appear as part of a syndromic condition or as an isolated malformation [2]. Currently, at least six different chromosomal loci have been identified for SHFM [3,4]. SHFM1 is the most common form of ectrodactyly, characterized by autosomal dominant inheritance, incomplete penetrance and variable expressivity. It is associated with hearing loss in 35% of affected individuals and has been variably associated with developmental delay/intellectual disability (DD/ID) and craniofacial anomalies. The recognition of translocations, deletions and inversions on chromosome 7q21.3 [5,6] has led to the identification of the SHFM1 locus, which includes the genes *DLX5* and *DLX6* [7,8,9,10]. In a mouse model, simultaneous knockout of *Dlx5* and *Dlx6* causes a phenotype overlapping with human ectrodactyly, as well as craniofacial and inner ear defects, thus demonstrating their fundamental role in embryonic development and particularly in limb formation [11].

Single-nucleotide variants in the *DLX5* gene have also been identified as a cause of SFHM1. An ectrodactyly phenotype with hearing loss was reported by Shamseldin et al. as an autosomal recessive trait in siblings with homozygous missense variants in the DNA binding domain of *DLX5* [12]. Split foot was then reported in a family with a heterozygous *DLX5* missense variant transmitted in an autosomal dominant manner; in their study, however, the authors performed a luciferase assay, which showed similar results for the variant they identified compared to the variant that was found in the homozygous state in the family by Shamseldin et al.; therefore, they hypothesized the presence of a second alteration they could not detect, either in *DLX5*/6 or in a regulatory element, as a contributing factor [13]. Autosomal dominant transmission has been reported in two unrelated families by Sowinska-Seidler et al. [14], sharing the same truncating variant of the *DLX5* gene; affected family members presented with isolated SHFM (without hearing loss, intellectual disability or craniofacial anomalies) with incomplete penetrance; the authors once again hypothesized the presence of a second alteration (perhaps a common polymorphism in a regulatory element) that could explain the reduced penetrance of the limb defect. A family with ectrodactyly caused by a missense variant in the *DLX6* gene has also been reported, and the phenotype was transmitted in an autosomal dominant fashion [15].

In the following years, 7q21 deletions not including the *DLX5*/6 genes, but at least partially including the gene *DYNC1I1* (located proximally to *DLX5*/6 on chromosome 7), have also been associated with the SHFM phenotype. The *DYNC1I1* gene itself is not expressed in the developing limb; however, Birnbaum et al. [16] demonstrated the presence of a physical interaction between exons 15 and 17 of *DYNC1I1* and the *DLX5*/6 genes, suggesting that these sequences act as exonic enhancers (eExons), thus regulating the expression of *DLX5*/6 and playing a major role in limb development [9,17]. *DYNC1I1* and *DLX5/6* are thought to interact through enhancer–promoter DNA looping, suggesting that *DYNC1I1* eExons 15–17 act as cis-acting enhancers [16]. The deletion or physical separation of the *DYNC1I1* eExons from the *DLX* genes (through translocation or inversion when at least one breakpoint separates the enhancers from the promoters) was demonstrated to be responsible for several cases of SHFM1.

In this study, we identified through SNP array a de novo 7q21 deletion involving exons 15 and 17 of *DYNC1I1,* but not including *DLX5*/6, in a patient affected by SHFM, bilateral deafness and an abnormality of the inner ear. We then performed an expression study of the *DLX5* and *DLX6* genes on a lymphoblastoid cell line (LCL) derived from our patient, which demonstrated reduced expression of both genes.

## 2. Materials and Methods

### 2.1. Karyotype and FISH Analysis

Standard chromosome analysis at 550-band resolution was carried out on metaphases obtained via PHA-stimulated circulating lymphocytes and GTG-banded. Locus-specific probes for FISH analysis were selected based on their genomic position from the clones library 32K (32K Library; BACPAC Resources, Oakland, CA, USA). DNA was extracted using the Quantum Prep MiniPrep Kit (BioRad, Hercules, CA, USA) and was SpectrumGreen-dUTP or SpectrumOrange-dUTP labeled using the Nick Translation kit (Abbott, Abbott Park, IL, USA) according to the manufacturer’s protocol.

### 2.2. SNP Array

Copy Number Variations Analysis was performed using the Cytogenetics Whole-Genome 2.7M array (Cyto2.7M array), consisting of 400,000 SNP probes and ~2.1 million copy number probes at an average probe spacing of 1395 bp [18]. Extracted DNA was digested, ligated and labeled following the manufacturer’s protocol (Affymetrix Inc., Santa Clara, CA, USA). Data analysis was conducted using the Chromosome Analysis Suite (ChAS) software (ThermoFisher Scientific, Waltham, MA, USA) using the control dataset provided by the manufacturer. All the duplications and deletions with a size greater than 75 Kb and including at least 25 probes were considered.

### 2.3. Lymphoblastoid Cell Line Establishment

Epstein–Barr virus (EBV)-transformed LCLs were established from patient’s and parental whole blood in lithium heparin layered onto Ficoll and centrifuged for 20 min; the lymphocyte layer was removed, washed with RPMI medium and resuspended with the filtered supernatant of B95.8 cell cultures (producing EBV virus). After an overnight incubation, the lymphocytes were resuspended in RPMI medium containing fetal bovine serum (FBS; 10%) and antibiotics and left for 14 days after which they were transformed.

### 2.4. Expression Study

RNA was extracted from lymphoblastoid cell lines using a commercial kit (RNeasy MINI Kit, Qiagen, Valencia, CA, USA). cDNA was obtained via retrotranscription of 1µg RNA with random hexamers according to the protocol of SuperScript IV (ThermoFisher Scientific). qPCR was performed in triplicate on each sample using an ABI 7900 Sequence Detection System (Applied Biosystems, Foster City, CA, USA) and DNA-binding dye SYBR Green (Invitrogen Corporation, Carlsbad, CA, USA) using GUSB as a reference gene. Specific primers were selected using PrimerExpress as follows: DLX5, fw-GCTGGGATTGACACAAACAC and rev-AGGCACCATTGAAAGTGTCC; DLX6, fw-TCGCTTTCAGCAGACACAGT and rev-CGGCTTCTTGCCACACTTAT; GUSB, fw- GAAGCCCATTATTCAGAGCGAGTA and rev-CTTCAGTGAACATCAGAGGTGGAT. The 2^−ΔΔCt^ comparative method was used to calculate DLX5 and DLX6 expression [19], treating the parental LCLs as healthy controls.

## 3. Results

### 3.1. Clinical Report

Our patient is the first-born son to healthy non-consanguineous parents. Pregnancy was unremarkable until the 32nd week, when fetal growth delay was observed. There were no known exposures to potential teratogens. Vaginal delivery was induced at the 37th week of gestation due to poor fetal growth. The parameters at birth were as follows: weight was 2100 g (3rd centile), length was 46 cm (10th centile), head circumference was 30 cm (<3rd centile, −2.7 SD) and Apgar score was 9 at 1′ and 9 at 5′. A newborn hearing screening with otoacoustic emissions was failed in both ears, and therefore the infant was referred to specialists for audiological evaluation. Repeated examinations and acoustic-evoked potentials confirmed asymmetrical bilateral severe–profound hearing loss, which was worse in the left ear, in which the V wave was not detectable. At the age of 4 months, hearing aids were applied. His developmental milestones were slightly delayed; he was able to sit without support at the age of 9 months and started walking at 18 months. He uttered his first words at 17 months of age.

The boy came to our attention at the age of 11 months; his length was 67.5 cm (<3rd centile, −2.6 SD), weight was 6600 g (<3rd centile, −3.5 SD) and head circumference was 41 cm (<3rd centile, <−4SD). On our last evaluation, the boy was 9 years and 9 months old; his height (119 cm), weight (23 kg) and head circumference (46.5 cm) were all below the third centile. He had slight brachycephaly, mild synophrys, a small nose with anteverted nostrils, slightly arched upper lip, raised palate, normal ears and sinus pilonidalis. He had a bilateral split hand with absence of the second and third fingers and 4th–5th partial cutaneous syndactyly, and bilateral split foot with absence of the second and third toes, 4th–5th cutaneous partial syndactyly, lateral deviation of terminal phalanx of the first toe and medial deviation of terminal phalanx of fifth toe bilaterally.

**Figure 1:**
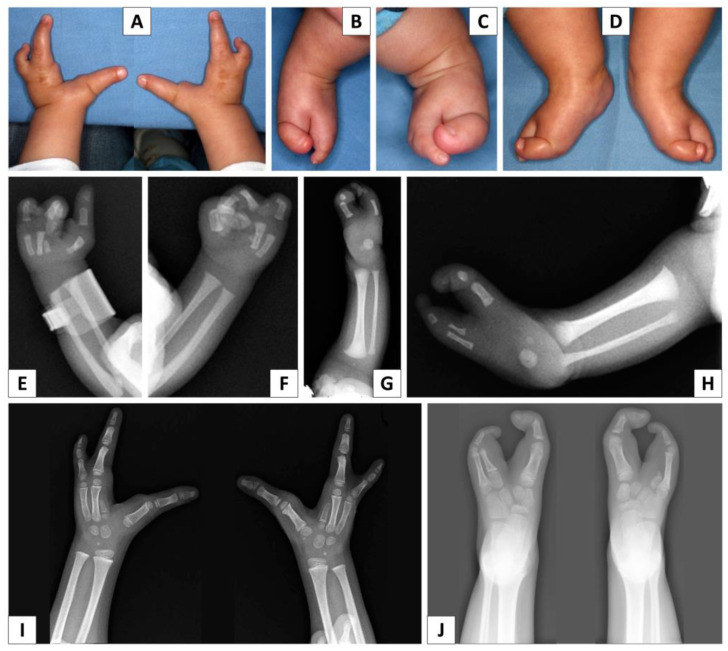
Photographs and X-ray of the hands (**A**,**E**,**F**,**I**) and feet (**B**–**D**,**G**,**H**,**J**) of the proband, showing bilateral SHFM.

X-rays of both hands showed the presence of the first, fourth and fifth metacarpal bones, the presence of the phalanges of the corresponding fingers and the presence of a little central hypoplastic metacarpal bone (Figure 1). Bone age was delayed (between 7 and 8 years with a chronological age of 9 years and 9 months). X-rays of both feet showed the presence of the first and fifth metatarsal bones, the presence of the phalanges of the corresponding toes and the presence of a little central hypoplastic metatarsal bone only in the left foot. Spine X-rays revealed a slight left convex deviation of the spine with regular shape of the vertebral bodies.

**Figure 2:**
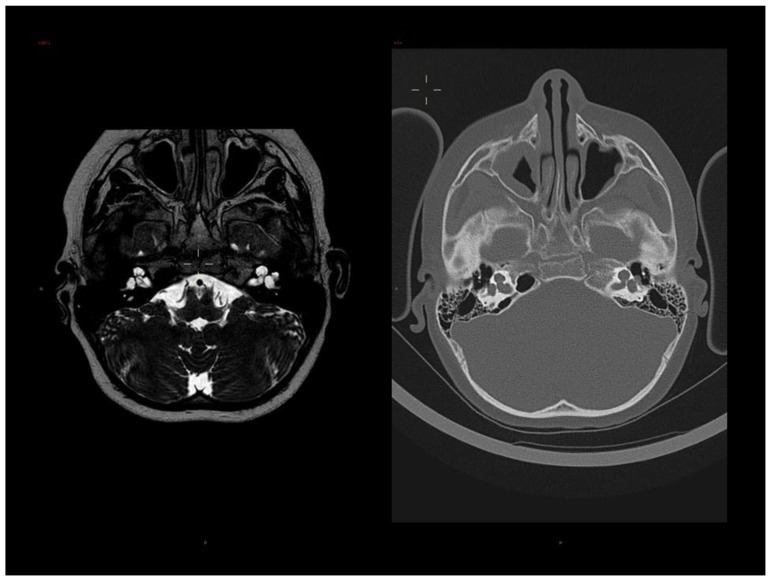
Brain MRI and CT of the proband, showing bilateral incomplete partition type I with defective internal structure of the cochlea and mildly enlarged vestibule on both sides, especially on the left, where a focal herniation of perilymphatic fluid through discontinuity of the bone wall at the stapedo-ovalar junction is observed. Mild enlargement of the right vestibular acqueduct and of the lateral semicircular canals.

During follow-up, at the age of 9 years and 9 months, because of progressive worsening of hearing on the left side, he underwent surgery for a left cochlear implant. Ear tomography and MRI performed before surgery showed incomplete partition type I with an enlarged cochlea and vestibule on both sides (Figure 2). Incomplete partition has been previously described in patients with SHFM1 [6,7,20,21,22,23]. In the anatomic seat of the stirrup, a herniation of the perilymphatic membrane through a discontinuity of the bone wall was observed. The lateral semicircular canals appeared mildly and uniformly dilated, in the absence of dehiscence. The lateral portion of the internal ear canal appeared ectasic on both sides. Therefore, cochlear implant surgery was implemented with repair of the peri-lymphatic hernia of the oval window. Brain MRI also revealed a mild verticalization of the anterior portion of the right collateral sulcus, a slightly malrotated aspect of the hippocampus resulting in a slight dimensional asymmetry of the temporal horns of the lateral ventricles.

The last neuropsychiatric evaluation (performed at age 9 years and 7 months) was normal, except for some phonological distortions and limitations in walking due to physical malformations. The following exams were normal: ECG, echocardiography, hip ultrasonographic evaluation, abdominal ultrasounds, brain ultrasounds, skin ultrasound for sinus pilonidalis and ophthalmological evaluation.

### 3.2. Molecular Analyses

**Figure 3:**
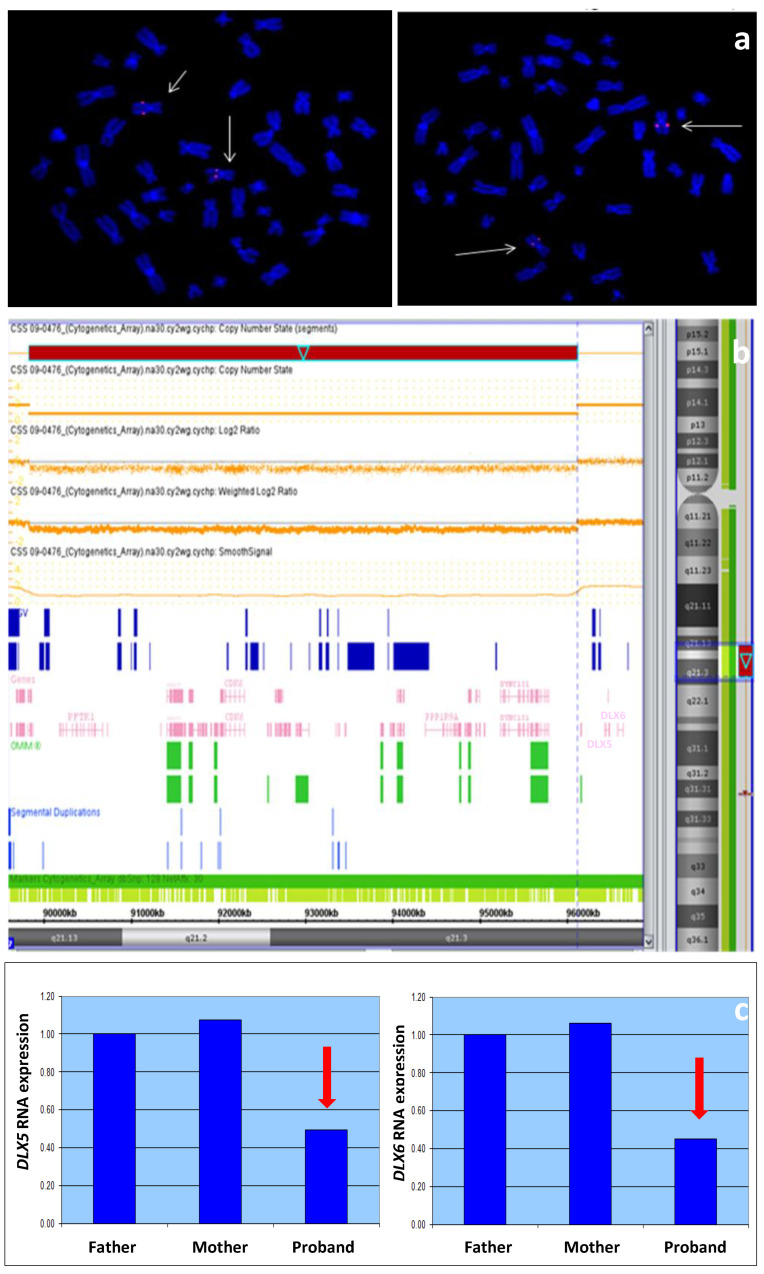
(**a**) Locus-specific FISH with N0002N02 (**left**) and N0418K11 (**right**) clones showing normal hybridization pattern (pink signals) on both chromosomes 7 (arrows). (**b**) SNP array analysis showing a deletion (red bar) of about 6.3 Mb at 7q21.13q21.3 proximal to *DLX5* and *DLX6* that encompasses 70 Refseq genes including *DYNC1I1* (black arrow). (**c**) *DLX5/6* expression calculated on RNA extracted from lymphoblastoid cell lines of the patient and his parents, showing a reduction in the patient’s sample (red arrows).

The karyotype was normal (46,XY). FISH analysis performed using SHFM1 locus-specific probes was negative. SNP array revealed a de novo microdeletion in the chromosomal region 7q21.13q7q21.3, spanning approximately 6.3 Mb from nucleotide 89,993,838 to 96,278,971 (hg19 release). The deletion, proximal to but not comprising the *DLX5* and *DLX6* genes, included *DYNC1I1* and other 67 genes (DECIPHER; https://www.deciphergenomics.org/patient/370310; accessed on 6 June 2023), which may have contributed to the phenotype. Since *DLX5/6* expression is regulated by eExons 15 and 17 of the *DYNC1I1* gene, to confirm the pathogenicity of the deletion we performed an expression study of the *DLX5* and *DLX6* genes through RT-PCR on LCLs derived from the family trio. As expected, the expression of both genes in the proband-derived LCL was reduced to about 40–45% compared to the non-deleted healthy parents (Figure 3).

## 4. Discussion

Correct embryonic development relies on the spatial and temporal differential expression of many genes implicated in embryogenetic processes. These complex gene interactions are finely controlled by regulatory elements, which can physically interact with target genes located even hundreds of kb away, thanks to the formation of chromatin loops within the functional conformations of the genome, defined as topologically associating domains (TADs) [24]. Molecular abnormalities such as CNVs (Copy Number Variations) involving conserved non-coding elements (CNEs), or balanced translocations separating CNEs from their target genes, can have a great impact on embryonic development, and have been associated over time with congenital malformations [25,26].

**Figure 4:**
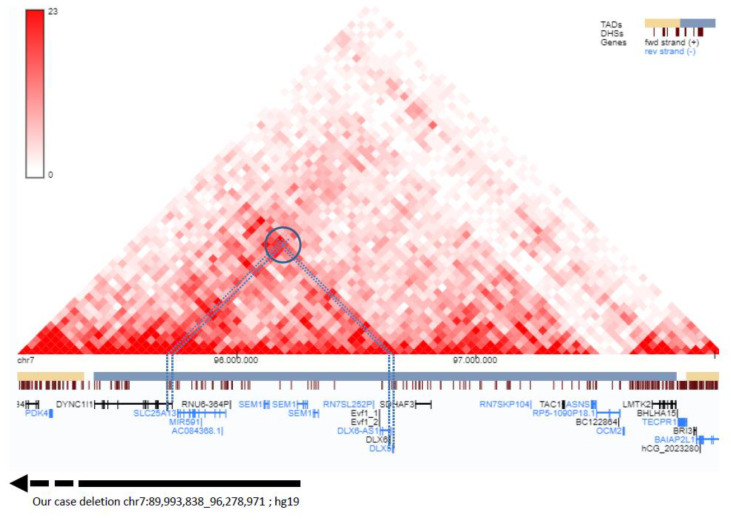
Detail of the distal portion of deletion (black line and arrow) and Hi-C map with TADs organization showing high grade of interactions (bright red dots) among the distal part of *DYNC1I1* and *DLX5/6* genes (dotted lines and circle). These data confirm that *DYNC1I1* exons 15 and 17 act as enhancers of *DLX5/6* by means of a physical interaction. The deletion includes the proximal TAD boundary and *DYNC1I1,* but not *DLX5/6* genes. The Hi-C map was visualized on 3D Genome Browser, based on data provided from GM12868 cell line by Rao et al., 2014. (http://3dgenome.fsm.northwestern.edu/view.php; last access 6 June 2023).

**Figure 5:**
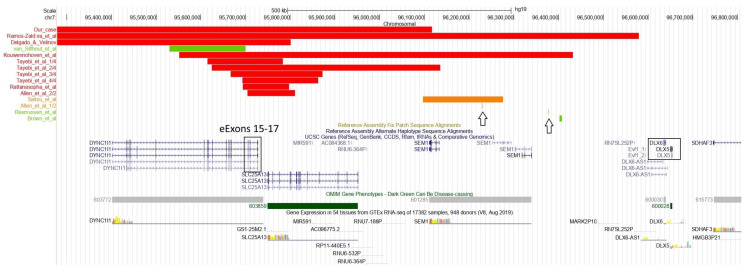
Chromosomal alterations of previously reported cases [https://genome.ucsc.edu/ GRCh37/hg19 (accessed on 23 June 2023)] [6,20,21,27,28,29,30,31,32,33]. Deletions are represented in red, inversions in green and translocations in orange; rearrangements with a single breakpoint or breakpoints that are very close to each other are highlighted with an arrow. Note the position of *DLX5/6* and that of eExons 15–17 of *DYNC1I1*. The *SLC25A13* gene, included in some of the deletions, is correlated to an autosomal recessive disease.

The TAD of the SHFM1 locus on chromosome 7 includes the *DYNC1I1* gene and its exons 15 and 17, which act as enhancers on the limb-expressed *DLX5*/*6* genes, and therefore play a significant role in limb development, as documented by Hi-C data showing a high grade of interactions between these genes (Figure 4). To date, deletions involving eExons 15 and 17 of *DYNC1I1,* not including *DLX5/6,* have been described in nine families [27,28,29,30,31,32]. Five cases with translocations/inversions with a breakpoint separating *DLX5*/6 and exons 15/17 of the *DYNC1I1* gene have also been reported [6,20,21,29,33]. In two families [21,32], affected members showed hearing loss and craniofacial abnormalities or neurodevelopmental disorder without the ectrodactyly phenotype. In the other published families, affected members presented with a SHFM phenotype with variable expressivity in the number and severity of the limb involvement, and some of the patients with split hand/foot also had associated features such as hearing impairment, developmental delay and/or intellectual disability and craniofacial alterations (see Table 1, Table 2 and Figure 5 for detailed description). A genotype–phenotype correlation has been suggested [20], but predicting clinical characteristics from molecular features is often unfeasible due to variable expressivity.

We present the case of a patient with a 6.3 Mb deletion in 7q21.13-q21.3 that involves part of SHFM1 TAD including the proximal boundary and eExons 15 and 17 of *DYNC1I1* but not the genes *DLX5* and *DLX6* (Figure 4), presenting with a SHFM phenotype and hearing loss associated with an abnormality of the inner ear (incomplete partition type I). Of the other 67 genes that were included in our patient’s deletion, there was no gene that could single-handedly be responsible for one or more of our patient’s features; however, we cannot exclude that their deletion may have contributed to his phenotype. We performed a review of the literature to collect information on the phenotype of patients with *DYNC1I1* deletion not including *DLX5*/6 or with translocations separating the eExons from their target genes.

The reduced expression of both *DLX5* and *DLX6* in LCLs derived from our proband confirms the etiology of the split hand/foot phenotype. Therefore, this case provides additional evidence concerning the fundamental role of *DYNC1I1* eExons 15 and 17 in limb development and that the loss of interaction between *DYNC1I1* eExons 15 and 17 and DLX5/6 is sufficient to manifest SHFM. Our results exemplify the role of CNVs and balanced chromosomal rearrangements involving regulatory elements in the etiology of congenital malformations.

Interestingly, on a different (osteoblastoid) cell line derived from a patient carrying a *DYNC1I1* deletion not including *DLX5/6*, Rattanasopha et al. [28] found absent expression of the *DLX5*/6 genes. To explain their result, Rattanasopha et al. hypothesized that *DLX5*/6 were maternally imprinted in osteoblasts, and, therefore, only the paternal allele was expressed. The authors based this hypothesis on the findings of two previous studies claiming that *DLX5* is imprinted in humans [34,35]. In the first of these studies, Okita et al. [34] used human–mouse monochromosomal hybrids to perform polymorphic analyses and concluded that *DLX5* is paternally imprinted (maternally expressed) in lymphoblasts and brain tissues. In a subsequent work, Horike et al. [35] supported the imprinting hypothesis, stating that *DLX5* expression is regulated by the *MECP2* gene and that *DLX5* is paternally imprinted in human lymphoblasts and brain tissues. They also stated that patients with Rett syndrome (caused by heterozygous mutations in the *MECP2* gene) exhibit loss of *DLX5* imprinting. However, it is still controversial whether the *DLX5* gene is really imprinted in humans [36]. In particular, a work from Schule et al. [37] from 2007 replicated the experiments performed by Horike et al. with very different results: their data showed that *DLX5* is not imprinted in human lymphoblasts and brain tissues, and that it is not a direct target of *MECP2* modulation. Another study by Itaba-Matsumoto et al. [38] failed to replicate the results obtained by Horike et al. concerning the relationship between *MECP2* and *DLX5* and concluded that they could not prove the existence of a correlation between the two genes. Horike’s group [39] responded with a letter to the editor, in which they repeated their experiments and replicated their previous results, again supporting the hypothesis that *DLX5* is imprinted in humans. Subsequently, a different group [40] published a map of Differentially Methylated Regions of chromosome 7 and did not find any in or near *DLX5* that would indicate imprinting.

The theory that the *DLX5*/6 genes are imprinted in humans, at least in peripheral blood cells, does not seem to be completely compatible with the 40–45% RNA level we detected in the patient-derived LCL compared to controls. If these genes were maternally imprinted in peripheral blood cells, as previously hypothesized [34], we could expect two alternative scenarios in the affected proband: (i) if our patient’s deletion was on the maternal allele (imprinted/not expressed), *DLX5*/6 RNA levels would be similar to those of controls, and we would not expect an SHFM phenotype (only the normal—paternal—allele would be expressed); (ii) if the deletion was on the paternal allele (expressed), we would expect absent RNA levels of the *DLX5/6* genes as reported in osteoblasts by Rattanasopha et al., since the paternal copy is lacking its upstream enhancers and cannot be normally expressed. Therefore, our data seems to support the notion that, at least in human-derived lymphoblasts, *DLX5*/6 are not imprinted, as previously stated by Schule et al. [37]. We cannot exclude that the *DLX5/6* genes are imprinted in different tissues or at different stages of embryonic development.

Our study has some obvious limitations. Since the proband was unavailable for additional testing, we could only study *DLX5/6* expression in the lymphoblastoid cell line; future studies could explore this matter further through evaluating *DLX5/6* expression in different tissues. We recognize that the immortalization of the lymphoblastoid line may cause changes in methylation patterns, that *DLX5*/6 are weakly expressed in lymphoblasts and that RT-PCR may not allow a precise quantification of transcript levels. However, segregation data from the literature show that there are different cases in which the alteration in the *DLX5* gene and/or regulatory element has been inherited from the mother [13,14,20,30], supporting the hypothesis that *DLX5*/6 are not maternally imprinted in humans.

## 5. Conclusions

This study supports the notion that chromosomal abnormalities involving regulatory elements can be responsible for congenital malformations through altering the relationship between enhancers and their target genes. Our results disagree with an imprinting of the SHFM1 locus in humans, at least in lymphoblasts. Although we cannot exclude that the *DLX5*/6 genes are paternally or maternally imprinted in different tissues, segregation data from families in the literature suggest equal transmission from both parents, arguing against a parent-of-origin effect in the recurrence of the ectrodactyly phenotype. The study of this locus and its regulation could serve as a model to help shed light on the complex mechanisms involved in embryological development.

## Figures and Tables

**Table 1:** Phenotype of patients described in the literature with disruption of *DYNC1I1* regulation without deletion of *DLX5/6*.

ID	Nr of Affected Relatives	Limb Anomalies	Neurodevelopment	Hearing Loss	Inner Ear Abnormality	Other
Van Silfhout et al. [6]	1	1 (bil SHFM)	1 (PDD NOS)	0	0	AVM right hand
Kouwenhoven et al. [27]	1	1 (bil SFM)	0	0	0	
Brown et al. [21]	5	0	2/5	1	1	Craniofacial abnormalities
Rattanasopha et al. [28]	6 (and 2 unaffected carriers)	1 (variable)	0	0	0	Right hand polydactyly (1/8)
Allen et al. 1/2 [29]	1	1 (variable)	0	1 (sensorineural deafness)	0	
Allen et al. 2/2 [29]	2	1 (variable)	0	0	0	
Tayebi et al. 1/4 [30]	3	1 (bil SHFM)	0	0	0	
Tayebi et al. 2/4 [30]	5	1	0	1 (severe HL)	0	
Tayebi et al. 3/4 [30]	6	1	0	0	0	
Tayebi et al. 4/4 [30]	2 (monozygotic twins)	1	0	0	0	
Delgado & Velinov [31]	4	1	1 (ID/DD)	0	0	
Rasmussen et al. [20]	5 (2 available for clinical examination)	1	2/2 (autism)	1	1	Craniofacial abnormalities
Ramos-Zaldìva et al. [32]	1	0	1 (paranoid personality disorder)	1	0	Dysmorphic features
Saitsu et al. [33]	1	1	1 (DD)	1 (severe HL, mixed type)	0	
Our case	1	1	1 (mild DD)	1	1	Poor growth

SHFM = split hand/foot malformation, SFM = split foot malformation, PDD NOS = pervasive developmental disorder not otherwise specified, ID = intellectual disability, DD = developmental delay, HL = hearing loss, AVM = arteriovenous malformation.

**Table 2:** Genotype of patients described in the literature with disruption of *DYNC1I1* regulation without deletion of *DLX5*/6.

ID	Karyotype	Type of Alteration	Deletion Size (kb)	Proximal bp	Distal bp	*DLX5*/*6* Deleted	eExons of *DYNC1I1* Deleted
van Silfhout et al. [6]	46,XY,inv(7)(p22q21.3)	Inversion		~95,530,000	~95,700,000	No	No
Kouwenhoven et al. [27]		Deletion	880	~95,552,000 *	~96,432,000 *	No	Yes
Brown et al. [21]	46,XX,inv7(q21.3q35) or 46,X,inv7(q21.3q35)	Inversion		96,401,902	96,407,985	No	No
Rattanasopha et al. [28]		Deletion	104	95,694,099	95,797,866	No	Yes
Allen et al. 1/2 [29]	t(2;7)(p25.1;q21.3)	Translocation		96,229,309	96,229,309	No	No
Allen et al. 2/2 [29]		Deletion	106	95,704,812	95,810,747	No	Yes
Tayebi et al. 1/4 [30]		Deletion	167	95,615,187	95,783,313	No	Yes
Tayebi et al. 2/4 [30]		Deletion	510	95,624,825	96,135,521	No	Yes
Tayebi et al. 3/4 [30]		Deletion	205	95,667,046	95,872,044	No	Yes
Tayebi et al. 4/4 [30]		Deletion	169	95,693,341	95,862,369	No	Yes
Delgado & Velinov [31]		Deletion	1032	94,769,383	95,801,045	No	Yes
Rasmussen et al. [20]	46,XX,inv(7)(q22q33)	Inversion		96,378,046	96,378,047	No	No
Ramos-Zaldìva et al. [32]	46,XY	Deletion	3190	93,389,222	96,579,845	No	Yes
Saitsu et al. [33]	46,XX,t(7;15)(q21;q15),t(9;14)(q21;q11.2)	Translocation		96,097,195	96,276,197	No	No
Our case	46,XY	Deletion	6300	89,831,744	96,116,907	No	Yes

* originally hg18; converted to hg19 using Lift Genome Annotations [https://genome.ucsc.edu/cgi-bin/hgLiftOver (accessed on 2 May 2023)]. For translocations or inversions, the coordinates reported here refer to the breakpoints involving chromosome 7 and closest to the SHFM1 locus.

## Data Availability

The data that support the findings of this study are available from the corresponding author upon reasonable request.
